# Supplementary material for: Empirical methods for controlling false positives and estimating confidence in ChIP-Seq peaks
Source: BMC Bioinformatics. 2008 Dec 5;9:523. doi: 10.1186/1471-2105-9-523 (PMC2628906; doi:10.1186/1471-2105-9-523)
Supplement: Additional File 1 — A variety of html documents from the USeq web site detailing the available applications, their best usage, output file type descriptions, command line menus, etc. [file 1471-2105-9-523-S1.zip › USeqUserGuides/JavaDoc.zip!/JavaDoc/gata/geneGlyphs/Annotation.html]

Annotation


|  |  |  |
| --- | --- | --- |
| **Overview** \| **Package** \| **Class** \| **Use** \| **Tree** \| **Deprecated** \| **Index** \| **Help** | |  |
| PREV CLASS   **NEXT CLASS** | **FRAMES**    **NO FRAMES**     **All Classes** |  |
| SUMMARY: NESTED \| FIELD \| CONSTR \| METHOD | DETAIL: FIELD \| CONSTR \| METHOD |  |


---


## gata.geneGlyphs Class Annotation

```
java.lang.Object
  gata.geneGlyphs.Annotation
```

---

``` public class Annotation extends java.lang.Object ```

**Author:**
:   nix
    Contains methods and fields to construct annotation glyphs.

---

**Constructor Summary**

`Annotation(AnnoSpecParams annoParams,
GATAParams gataParams)`


| **Method Summary** | |
| --- | --- |
| `void` | `assignGenericFeatureTracks()` |
| `void` | `colorizeTrackedGenericGlyphs(int converter)`             This method converts scores in generic glyphs to changes in alpha, the opacity number for the glyph color. |
| `void` | `drawDryRunAnnotation()` |
| `void` | `drawGenericGlyphs()`             Call each time parameters effecting GenericGlyphs change |
| `void` | `drawGlyphs()`             Call each time the scale changes or user alters a glyph viewablility |
| `GlyphPanel` | `getGlyphPanel()` |
| `int` | `getTrackConverter()` |
| `void` | `initializeTrackLabels()` |
| `void` | `makeGenericGlyphs()`             This makes a sorted array of GenericGlyphs broken down by track |
| `void` | `makeGlyphs()`             Call once to make the glyphs used in rendering the gene annotation. |
| `void` | `redrawAnnotation()` |
| `void` | `setAnnotationBackground()` |
| `void` | `setDefaultTrackThickness()` |
| `void` | `setInitialTrackColors()`             Use to initialize all generic glyphs color field |
| `void` | `setTrackDefaults()` |

**Methods inherited from class java.lang.Object**

`equals, getClass, hashCode, notify, notifyAll, toString, wait, wait, wait`

**Constructor Detail**

### Annotation

```
public Annotation(AnnoSpecParams annoParams,
                  GATAParams gataParams)
```


**Method Detail**

### redrawAnnotation

```
public void redrawAnnotation()
```

---


### drawDryRunAnnotation

```
public void drawDryRunAnnotation()
```

---


### setAnnotationBackground

```
public void setAnnotationBackground()
```

---


### drawGenericGlyphs

```
public void drawGenericGlyphs()
```

:   Call each time parameters effecting GenericGlyphs change

---


### makeGenericGlyphs

```
public void makeGenericGlyphs()
```

:   This makes a sorted array of GenericGlyphs broken down by track

---


### colorizeTrackedGenericGlyphs

```
public void colorizeTrackedGenericGlyphs(int converter)
```

:   This method converts scores in generic glyphs to changes in alpha, the opacity
    number for the glyph color. The lower the score the lower the alpha. The trickie business is
    using different scales since sometimes the scores are in log or lnx units. 70 is
    the lowest alpha allowed, everything else normalized from 70 to 255

---


### setTrackDefaults

```
public void setTrackDefaults()
```

---


### setInitialTrackColors

```
public void setInitialTrackColors()
```

:   Use to initialize all generic glyphs color field

---


### setDefaultTrackThickness

```
public void setDefaultTrackThickness()
```

---


### initializeTrackLabels

```
public void initializeTrackLabels()
```

---


### assignGenericFeatureTracks

```
public void assignGenericFeatureTracks()
```

---


### drawGlyphs

```
public void drawGlyphs()
```

:   Call each time the scale changes or user alters a glyph viewablility

---


### makeGlyphs

```
public void makeGlyphs()
```

:   Call once to make the glyphs used in rendering the gene annotation.

---


### getGlyphPanel

```
public GlyphPanel getGlyphPanel()
```

---


### getTrackConverter

```
public int getTrackConverter()
```


---


|  |  |  |
| --- | --- | --- |
| **Overview** \| **Package** \| **Class** \| **Use** \| **Tree** \| **Deprecated** \| **Index** \| **Help** | |  |
| PREV CLASS   **NEXT CLASS** | **FRAMES**    **NO FRAMES**     **All Classes** |  |
| SUMMARY: NESTED \| FIELD \| CONSTR \| METHOD | DETAIL: FIELD \| CONSTR \| METHOD |  |


---
